# Utilizing machine learning to improve clinical trial design for acute respiratory distress syndrome

**DOI:** 10.1038/s41746-021-00505-5

**Published:** 2021-09-09

**Authors:** E. Schwager, K. Jansson, A. Rahman, S. Schiffer, Y. Chang, G. Boverman, B. Gross, M. Xu-Wilson, P. Boehme, H. Truebel, J. J. Frassica

**Affiliations:** 1 Philips Research North America, Cambridge, MA USA; 2 Research & Development, Pharmaceuticals, Bayer AG, Wuppertal, Germany; 3 Faculty of Health, Witten/Herdecke University, Witten, Germany; 4 Institute for Medical Engineering and Science, Massachusetts Institute of Technology, Cambridge, MA USA

**Keywords:** Drug development, Clinical trial design

## Abstract

Heterogeneous patient populations, complex pharmacology and low recruitment rates in the Intensive Care Unit (ICU) have led to the failure of many clinical trials. Recently, machine learning (ML) emerged as a new technology to process and identify big data relationships, enabling a new era in clinical trial design. In this study, we designed a ML model for predictively stratifying acute respiratory distress syndrome (ARDS) patients, ultimately reducing the required number of patients by increasing statistical power through cohort homogeneity. From the Philips eICU Research Institute (eRI) database, no less than 51,555 ARDS patients were extracted. We defined three subpopulations by outcome: (1) rapid death, (2) spontaneous recovery, and (3) long-stay patients. A retrospective univariate analysis identified highly predictive variables for each outcome. All 220 variables were used to determine the most accurate and generalizable model to predict long-stay patients. Multiclass gradient boosting was identified as the best-performing ML model. Whereas alterations in pH, bicarbonate or lactate proved to be strong predictors for rapid death in the univariate analysis, only the multivariate ML model was able to reliably differentiate the disease course of the long-stay outcome population (AUC of 0.77). We demonstrate the feasibility of prospective patient stratification using ML algorithms in the by far largest ARDS cohort reported to date. Our algorithm can identify patients with sufficiently long ARDS episodes to allow time for patients to respond to therapy, increasing statistical power. Further, early enrollment alerts may increase recruitment rate.

## Introduction

Although great progress in pharmaceutical therapies has been achieved over the last decades, some diseases, especially in the intensive care unit (ICU), are still characterized by high mortality, and efficient therapies would be well-received^1^. However, clinical trials in the ICU are highly complex and prone to failure. A prominent example is acute respiratory distress syndrome (ARDS). It is associated with a mortality of up to 40% and significantly reduced quality of life among survivors^2^. Among others, leading causes for ARDS are pneumonia, sepsis, or aspiration^3^. Three factors particularly stand out as reasons why so many trials in ARDS have failed, despite promising preclinical results, observational data, and clinical expert knowledge^4^:

1. *Heterogeneous patient populations*. Different aetiologies for ARDS, combined with diverse patient comorbidities, lead to different disease phenotypes that may require different treatment^4^. Additionally, ICUs vary in their standard of care, making it difficult to disentangle the effects of ICU practice and proposed therapies^1^.
2. *Different pharmacological modes of action*. In former clinical trials, too little attention was paid to patient stratification regarding the pharmacodynamic and pharmacokinetic characteristics of the therapeutic candidate. While fast-acting vasoactive drugs like inhaled nitric oxide might show a direct benefit on oxygenation^5^, immunomodulatory drug candidates, such as GM-CSF might show only a delayed effect and need more time to deliver their therapeutic potential^6^.
3. *Low recruitment rates*. ARDS itself is underdiagnosed and often detected too late, even in a clinical trial setting, with clinical recognition as low as 51%^2^. Additionally, at the time of ARDS diagnosis, physicians are faced with severely ill patients that are prone to instability. Inclusion of these patients into a clinical trial, although meaningful, is of less importance to care givers at the time of diagnosis^7^.

**Fig. 1:**
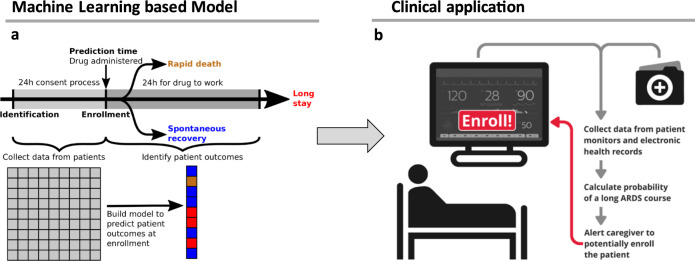
Overview of proposed model use case. Schematic illustration model building and patient stratification (a). Here, patients with a high probability of a long ARDS course will be predicted using data from both electronic health records and real-time patient monitoring. Caregivers can then be alerted when such a patient is identified (b).

According to Rilley et al., several studies regarding ARDS phenotypes have been performed, totaling about 20,000 patients^8^ (Supplementary Fig. 1). However, allocating patients to these phenotypes remains challenging, given the examinations needed. To overcome these challenges, we provide a new data-driven approach for clinical trial design and patient stratification using machine learning and artificial intelligence. We leveraged a database including data from over 700 intensive care units and more than 2.7 million individual patient stays over the calendar years 2002–2016^9^ to create a patient stratification algorithm for ARDS. We aimed to define suitable baseline criteria for patient inclusion into clinical trials by addressing the heterogeneity of disease courses and reducing the interpatient variability. By defining different patient subpopulations, we wanted to identify specific patients that might benefit from a particular therapy, whether it is e.g., fast acting (within 24 h) or one that has a delayed therapeutic onset. The idea of this approach was to create a highly flexible machine learning model (Fig. 1a) that can be incorporated into a simple, autonomous, and user-friendly clinical tool for quick and purposeful patient enrollment (Fig. 1b).

Such a tool can increase the statistical power and reduce the required sample size of prospective ARDS clinical trials.

## Results

### Study population

**Fig. 2:**
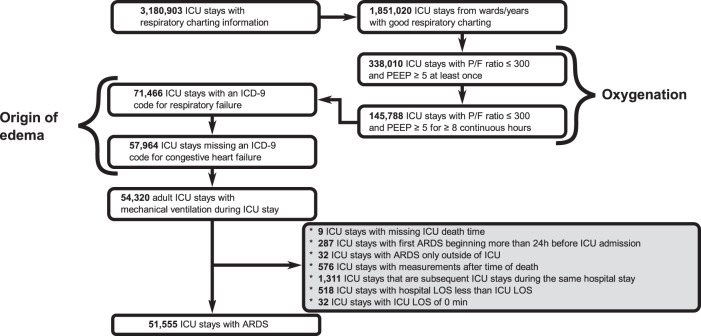
Data-driven extraction of ARDS cohort. The extraction of the ARDS cohort from the eRI database. From 3 million ICU stays in the database, we retained only mechanically-ventilated adults with poor oxygenation (defined as P/F ratio ≤ 300 and PEEP ≥ 5 for at least 8 h) and whose edema was due to respiratory failure rather than congestive heart failure, as captured by ICD-9 codes recorded during ICU stay.

From 3,180,903 ICU stays with respiratory charting information contained in the Philips’ eICU Research Institute (eRI) database, 51,555 ICU patients were identified as having ARDS defined by low oxygenation, respiratory failure with mechanical ventilation in the absence of congestive heart failure (Fig. 2; see “Methods” section).

**Fig. 3:**
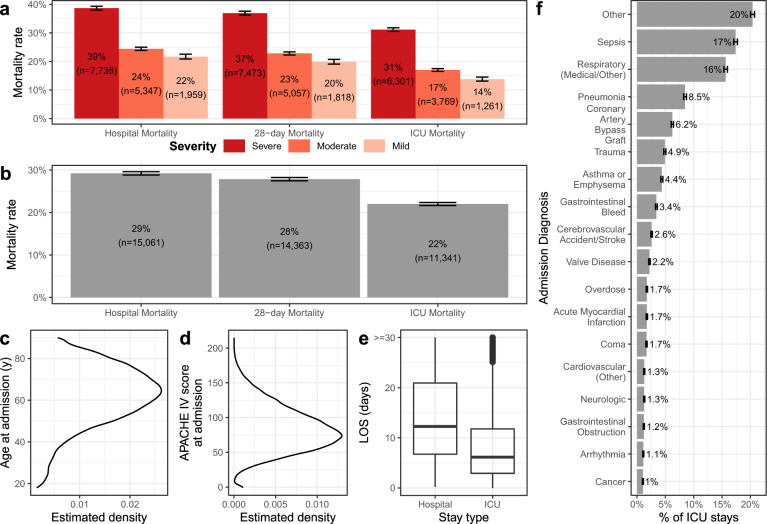
Characterization of ARDS cohort. The mortality rate by severity at onset (**a**), overall mortality rates (**b**), age (**c**), APACHE IV scores (**d**), lengths of stay (LOS) both in-hospital and in-ICU (**e**), and admission diagnoses (**f**). Severity was determined by the minimum *P*/*F* ratio in the first 8 h after ARDS onset. Error bars represent 95% confidence intervals. Panels **c**, **d** represent estimated densities. **e** The boxplot elements are: center line, median; box limits, upper and lower quartiles; whiskers, 1.5× interquartile range; points, outliers.

Hospital mortality among ICU patients diagnosed with severe ARDS (median P/F ratio in the first 8 h ≤100 mmHg) was 39%. In general, higher severity levels at ARDS onset were positively correlated with higher mortality rates (Fig. 3a). Overall hospital mortality for our ARDS cohort was 29%. Nearly all of them died within the first 28 days (28%), 22% were in ICU at the time of death (Fig. 3b). They had an average age of 61.86 (sd 15.46) years, an average APACHE IV score of 84 (sd 32) at admission, and spent an average of 8.9 (sd 9.5) days in the ICU (Fig. 3c–e). Common admission diagnoses reflected known ARDS risk factors, including pneumonia, sepsis, and shock (Fig. 3f). The median duration of ARDS in our cohort was 20.4 h (IQR 16.4–26.2 h) (Supplementary Fig. 2).

### Subpopulation characterization (univariate analysis)

**Fig. 4:**
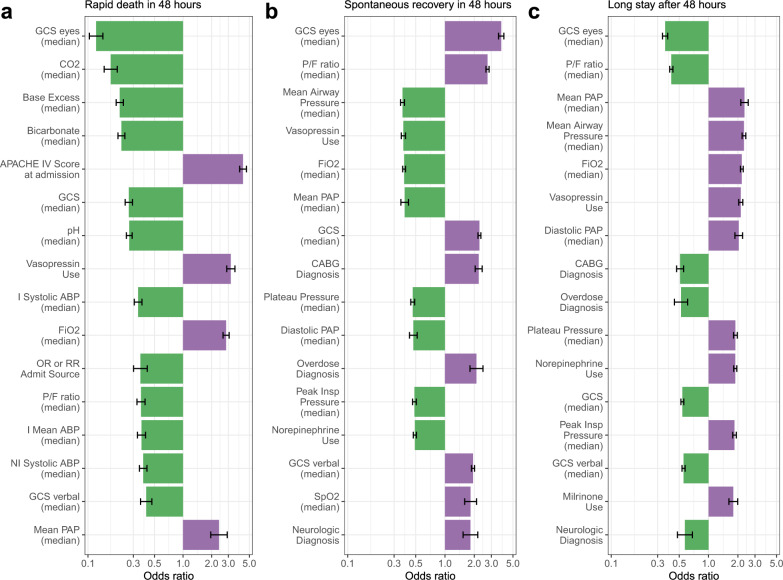
Top univariate associations with patient status 48 h after identification. Univariate logistic regressions were run associating each variable with three outcomes: rapid death vs. spontaneous recovery or long stay (**a**), spontaneous recovery vs. rapid death or long stay (**b**) long stay vs. spontaneous recovery or rapid death (**c**). Only the 15 significant features with the largest effects are plotted. Patients were categorized as rapid death (died within 48 h of identification), spontaneous recovery (had no ARDS after 48 h post-identification), or long stay (continued to have ARDS events after 48 h post-identification). Each time-varying predictor (e.g., PaCO_2_) was summarized over the first 24 h after identification using either the median or a missingness indicator (1 if measured, 0 if not). Each continuous predictor (e.g., median PaCO_2_ or age) was normalized by the difference in the 20th and 80th percentiles. Green bars indicate that higher variable values are associated with a lower risk of the outcome; purple bars indicate that higher variable values are associated with a higher risk of the outcome. CO2’s negative relationship with rapid death is due to its dependence on bicarbonate. Error bars represent 95% confidence intervals.

We used univariate logistic regression to characterize the factors distinguishing the subpopulations of rapid death, spontaneous recovery, or long stay patients (Fig. 4; see “Methods” section). A broad set of variables available from the eRI database, including static variables such as demographics, admission diagnoses, and admission sources, as well as dynamic variables such as hemodynamic and ventilatory parameters, vital signs, and laboratory measurements (see Supplementary Table 1) were leveraged. Importance of variables varied between the subpopulations. Interestingly, the Glasgow coma scale (GCS) eye score was the strongest predictor for all three sub-populations: a high score was negatively correlated with rapid death (odds ratio: 0.122) and a long stay after 48 h (odds ratio: 0.358), but positively correlated to spontaneous recovery (odds ratio: 3.79) (Fig. 4a–c). For patients who died rapidly, median values for CO_2_, base excess, and bicarbonate, between identification and enrollment, as well as high APACHE IV score at admission, were among the strongest predictors (Fig. 4a). High median values for *P*/*F* ratio, and low values for mean airway pressure were most predictive of a spontaneous recovery, while long stay patients showed the opposite pattern (Fig. 4b, c). Looking at the odds ratios, rapid death patients had the strongest, and long stay patients the weakest, univariate predictors.

**Fig. 5:**
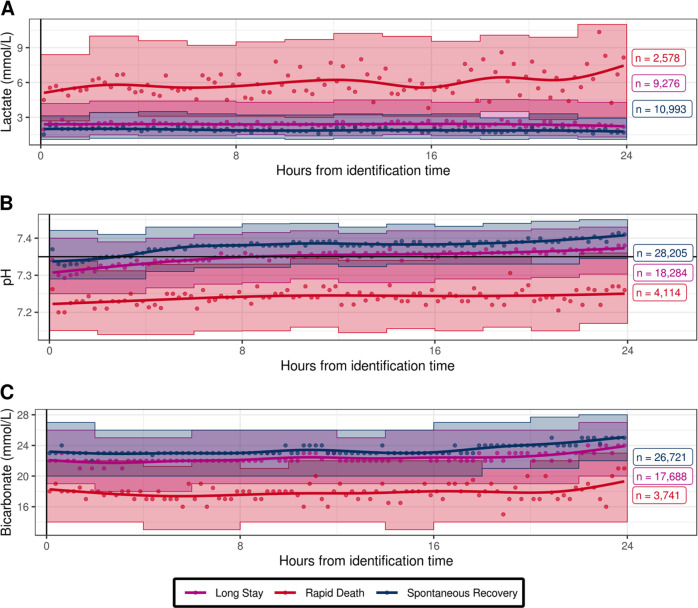
Trends of acidotic parameters over time by patient status 48 h after identification time. Smoothed trends between the identification time and 24 h afterwards by patient subtype for lactate (**a**), pH (**b**), and bicarbonate (**c**). Points represent the median parameter values in a quarter-hour interval relative to identification time. Intervals represent the 25th and 75th percentiles of parameter values in a 2 h window relative to identification time. GAM smoothing splines highlight trends. Number of subjects with plausible values in each category is shown on the right of each plot.

The worsening of metabolic acidosis was a distinguishing factor for rapid death patients, with high lactate, low pH, and low bicarbonate levels compared to spontaneous recovery or long stay patients (Fig. 5).

### Model building

Next, we evaluated twelve machine learning model architecture and framework combinations to determine which would be best to reliably predict patient outcome. Multiclass gradient boosting was selected for its performance and ease of interpretation.

Of the three architectures (random forest, gradient boosting, and logistic regression), gradient boosting had the highest consistent performance. Logistic regression had worse performance than the ensemble models. Gradient boosting and random forest achieved *F*-0.5 values on the internal validation set of 0.602–0.622, while logistic regression only achieved *F*-0.5 values of 0.563–0.579 (Supplementary Fig. 3). While gradient boosting had quite similar performance on the training and internal validation set (*F*-0.5 scores of 0.651–0.707 on training vs. 0.608–0.622 on internal validation), the performance of random forest decreased substantially (*F*-0.5 scores of 1 to 0.602–0.611).

All four model setups (three-way, three-way nested, two-way, and multiclass) used with gradient boosting had comparable performance on the internal validation set (*F*-0.5 scores of 0.608–0.622). The multiclass setup provides interpretable risk scores by giving each outcome a probability such that the sum of all three outcomes is one.

**Fig. 6:**
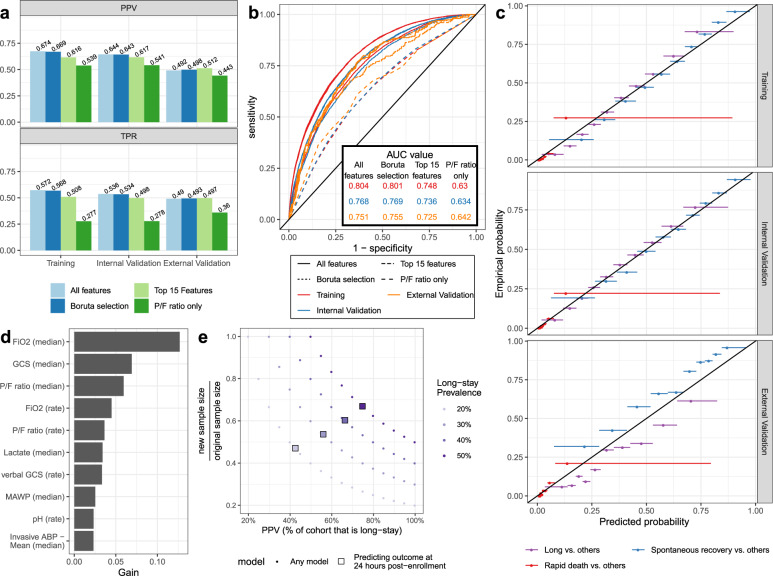
Predicting patients with a long ARDS course. The multiclass gradient-boosting models had strong performance on the internal and external validation datasets as measured by the positive predictive value (PPV) and true positive rate (TPR) (**a**). The receiver operating characteristics curve was calculated for identifying long stay vs. other patients (**b**; see “Methods” section). Model calibration was evaluated on the training, internal validation, and external validation datasets by binning the predicted probabilities into deciles and calculating the outcome rate among all patients with predictions in the given decile (**c**; see “Methods” section). The top ten features by gain are shown (MAWP mean airway pressure, GCS Glasgow coma scale; ABP arterial blood pressure) (**d**). The effect of the model on total sample size (**e**) can be substantial. Panels **c**–**e** display results from the Boruta-selected model.

Having selected the final model, we evaluated its performance and calibration on the internal and external validation sets. The model had strong performance on both datasets, achieving a positive predictive value (PPV) of 0.644 and true positive rate (TPR) of 0.536 on the internal validation set and a PPV of 0.492 and TPR of 0.490 on the external validation set (Fig. 6a). The area under the curve (AUC) for identifying long stay patients was 0.768 for the internal validation set and 0.751 in the external validation set (Fig. 6b; see “Methods” section for details). These represented substantial gains in performance over using a model trained using only *P*/*F* ratio (*P*/*F* ratio only model; Fig. 6a, b). We trained two additional models with feature subsets (see “Methods” section), a Boruta-selected model and a top-15-feature model; these demonstrated comparable performance to the original model with fewer features (106 and 15, respectively; Fig. 6a, b). In addition to good performance, the Boruta-selected model also had good calibration, which measures how well the predicted probabilities align with the actual outcome probabilities^10^. The model achieved nearly perfect calibration on both the training and the and internal validation datasets (Fig. 6c), with Brier scores of 0.126 and 0.135, respectively (Supplementary Table 5). Calibration on the external validation set was only slightly worse (Fig. 6c), with a Brier score of 0.125 (Supplementary Table 5).

The most important features of the Boruta-selected multiclass gradient boosting model were FiO2 levels and trends between identification and enrollment (Fig. 6d). GCS, pH, and lactate were also important discriminators. Examining these features between identification and enrollment, pH, lactate, and GCS are highly distinguishing of patients who die rapidly (Supplementary Fig. 4b–d). However, FiO2 levels are very different between the three subpopulations (Supplementary Fig. 4a): rapid death patients tend to have high FiO2 values, and spontaneous recovery patients low values. Further, the FiO2 values for spontaneous recovery patients decrease more quickly than those for long-stay ICU patients.

The ultimate goal of our predictive model is to reduce the necessary number of patients to enroll by enriching the enrolled population for patients with long ARDS durations. The level of enrichment depends both on the prevalence of long-stay patients in the ICU population and the positive predictive value (PPV) of the model (see “Methods” section). In our data, the prevalence of long-stay patients in a single hospital varied between 10 and 60% (Supplementary Fig. 5). Given the model’s PPV of 0.425 at 20% prevalence, up to a 50% reduction in sample size could be achieved with this model in practice (Fig. 6e).

## Discussion

Clinical trials of pharmacologic interventions for ARDS have been conducted for over 30 years. So far, none have shown significant benefit regarding primary endpoints in larger clinical studies, despite promising small single center clinical trials^1^. Inhaled nitric oxide and statins were both successfully tested in small study populations but failed in phase 3 studies^11,12^. Other unsuccessful pharmacologic therapies evaluated in ARDS patients are surfactant^13^, beta 2 adrenergic agonizts^14^, antioxidants^15^, ketoconazole^16^, lysofylline^17^, mesenchymal stem cells^18^, and omega-3 supplementation^19^. Problems of translatability from single center into large multicentre clinical trials might include different care standard among the centers as well as greater patient heterogeneity. Putting more effort into large-scale patient stratification seems to be key in improving translation, by identifying homogenous patient populations who might benefit from specific drug candidates^1^. This was recently highlighted by Kallet and colleagues, who reported that they found the response to aerosolized prostacyclin in ARDS to be highly dependent on hypoxemia and other factors^20^.

We aimed to develop a new data-driven approach for clinical trial design and patient stratification in the ICU using machine learning and artificial intelligence. Our goal was to build a predictive model to select ARDS patients, independent of the underlying etiology, who will have a sufficiently long disease course to benefit from a proposed therapy. Such a system would ensure that a patient recruited for a trial would likely still be in an ARDS state by the time the proposed therapy can affect the patient’s outcome. The model uses data from electronic health records and patient monitoring to automatically predict a patient’s future ARDS course (Fig. 1).

To demonstrate a possible application example for our approach, we defined subpopulations based on their prospective outcome 24 h after enrollment. If the drug candidate under investigation is known to have a delayed impact on the disease course, it is essential to only include patients into the trial that are predicted to live for a certain period, giving the drug a chance to display its potential.

To build our model, we leveraged data from more than 50,000 ICU stays with ARDS. The database was comprised of data from over 700 ICUs from across the United States, from both small rural hospitals and large urban teaching hospitals. ARDS severity and demographics (Fig. 3) within our cohort was very similar to that previously reported by Bellani and colleagues^2^. Higher mortality in the Bellani cohort may be due to the fact that Bellani’s study was conducted mainly during the winter months, whereas our ARDS cohort comprises patients from multiple years. This is also reflected in the ICU length of stay; on average, Bellani et al.’s patients spent a day longer on ICU than patients from the eRI database. The size of our cohort allows for higher resolution analysis, adds significant power to previous findings, and contributes to the confidence into our predictive AI model. On the other hand, large multiyear, multi-institution datasets hold a potential bias of unaccounted-for changes or variations in practice, particularly if these are associated with the number of ARDS patients identified. However, such variation is also a strength of our model, increasing the likelihood that it will generalize well across institutions with different protocols and care practices.

One interesting finding from the univariate analysis was that overdose, coronary artery bypass graft (CABG), and central-nervous-system-related admission diagnoses (like neoplasms or seizures, see Supplementary Table 3), were negatively associated with the long-stay ARDS disease course but positively associated with spontaneous recovery (Fig. 4b, c). This suggests that these indirect insults only have a transient effect on lung function, with a high probability for spontaneous recovery, making these patients unsuitable for drugs targeting ARDS in clinical trials. Another interesting finding was that elevated pulmonary artery pressure was negatively associated with spontaneous recovery but positively correlated with a long-stay outcome, consistent with the research performed by Calcaianu and colleagues^21^.

The time course of variables related to acid base disorders, drawn also from the univariate analysis (Fig. 5), shows that such disorders can help to prospectively identify patients that are prone to die quickly. These patients are unlikely to survive 24 h after trial enrollment, as metabolic impairment deviated distinctively from the other two subpopulations, confirming the importance of acid-base adaption variables to a persistent hypercapnia^22^. Further, this and the univariate analysis confirmed that long stay patients are “in between” spontaneous recovery and rapid death patients with respect to most measured parameters (Fig. 4). This suggested that linear or logistic regression would be ill-equipped to distinguish long stay patients from spontaneous recovery and rapid death patients (Supplementary Fig. 3). This problem, as well as the potential for correlated variables, was addressed by using more flexible ensemble approaches in our multivariable modeling.

Among twelve different models, a multiclass gradient boosting model combined good performance with interpretable risk scores (Supplementary Fig. 3). Its good performance on both the internal and external validation sets suggested that it can generalize well to other data sets (Fig. 6a, b). Boruta feature selection decreased the number of features from 220 to 106; while the top-15 feature model demonstrated a slight drop in performance, its small feature set might make it more suitable for future deployment. Further, the Boruta-selected model had nearly perfect calibration on the training and internal validation datasets (Fig. 6c and Supplementary Table 5), and only slightly worse calibration on the external validation dataset. This is expected since the external validation set comes from a different patient population than the training and internal validation sets (Supplementary Fig. 6). Although the Hosmer–Lemeshow test results demonstrate that the model’s predicted probabilities are different from the observed probabilities (Supplementary Table 6), the Brier scores^23^ and estimated calibration indices^24^, which measure the *degree* of discrepancy, are still low (Supplementary Table 5). Good calibration on the external validation set suggests that the model’s predicted probabilities would generalize well to other patient populations.

Currently different groups are using soluble biomarkers to stratify ARDS patients. For instance, Calfee et al. found a hyper-inflammatory and a hypo-inflammatory biomarker cluster, which seem to respond differently to treatment strategies and are stable over several days^25^. However, the limited availability of point-of-care devices for biomarker and the measurement turn-around-time combined with the short time window, and disease dynamic in clinical trials in the ICU limits soluble biomarkers for clinical trial enrichment.

Our approach has several advantages. With the help of caretaker assist devices, medical staff can be alerted within 24 h of ARDS identification if a patient is eligible for a clinical trial because of the predicted disease course. While an earlier prediction of the disease course—e.g., at the time of ARDS diagnosis—would be desirable, it is recommended to base treatment-decisions on the suggested time frame, as the performance is considerably lower at the time of identification (Supplementary Fig. 7). The autonomy and high predictability of such an approach will help to translate more medicines from bench to bedside. Further, such a tool can itself improve the care of the patients by increasing the clinical recognition of ARDS, and highlighting the important factors that contribute to patients’ disease course.

The goal of our study was to build an illustrative predictive machine learning and AI-based model for patient stratification in ARDS clinical trials. Using data derived from real-time electronic health care records, we trained our model to prospectively identify a patient class that would have a sufficiently long disease course to benefit from a therapy. This model will help to overcome the challenges of a heterogeneous patient population, low and late recruitment, and will tailor the stratification to the needs of a certain drug candidate. Applying the model will be a step forward towards successful clinical trials in the ICU that are urgently needed to help to reduce the high mortality, as currently highlighted by the ongoing Covid-19 crisis^26^.

ARDS patient identification according to the Berlin definition would also comprise imaging as an integral part. We did not include imaging into our patient identification, as for many patients imaging was not available. We hypothesized images might not be available even if patients truly had ARDS^2^. While we consider it a strength of our model that ARDS patient identification and stratification was possible even without imaging, future approaches should aim to incorporate (automated) analyses of images such as x-ray or CT scans.

The variables included in our model also did not allow for treatment effects to be accounted for, except for changes in ventilator settings. ARDS-specific treatments such as proning were poorly documented and thus difficult to use. Future work could investigate the impact of specific established treatments on the disease courses of ARDS patients.

## Methods

### eRI database

The data used in this study came from the Philips eRI data repository with hospital discharge dates ranging from 2002 to 2016^27^. The multi-institution eRI database contains patient data from 2.7 million patients across 787 heterogenous US ICU wards, including academic medical centers, and large and small community hospitals. For those patients, more than 2.8 billion vital sign measurements, 100 million medication orders and 800 million lab values were available. All analyses were conducted after removal of patient and institutional identifying information under a waiver of the requirement to informed consent by the appropriate institutional review boards from participating institutions that contributed to the eRI database.

### ARDS cohort extraction and subpopulation stratification

ARDS patients were identified according to Fig. 2, using the Berlin criteria, comprising: acute onset, and *P*/*F* ratio <300 mmHg with a PEEP ≥ 5 cm H_2_O for at least eight continuous hours. For many patients no imaging data was available, so we did not include imaging in the systematic patient identification. While we excluded patients with congestive heart failure, we were unable to exclude patients with fluid overload. ARDS patients further were mechanically ventilated, had an ICD-9 for respiratory failure, no ICD-9 codes for congestive heart failure, and a minimum age of 18. Identification and enrollment times were extracted for each patient. Time of identification was either the ICU admission time, if a patient met the ARDS criteria at admission, or the start of the first ARDS episode post-admission. Enrollment was set as 24 h after ARDS identification time to reflect trial practice, where enrollment of an identified patient can take considerable time. All ARDS patients were stratified according to the following outcomes: “rapid death” if a patient expired within 24 h after time of enrollment (48 h after ARDS identification), “spontaneous recovery” if a patient fully recovered from ARDS within 24 h after time of enrollment, and “long stay” if a patient continued to have ARDS for more than 24 h of post-enrollment. Only one ICU stay was included for each patient.

### Predictor variable extraction

Static and time-varying variables (Supplementary Table 1) were extracted from eRI. Outliers were removed according to Supplementary Table 2. All non-missing values of time-varying variables measured in the 24 h between identification and enrollment were summarized in three ways^1^: The median value in the 24 h after identification^2^; The variance of values in the 24 h after identification (only calculated if at least two values were recorded); and^3^ The rate of change of the values in the 24 h after identification (only calculated if at least two values were recorded at separate times). Admission diagnoses were grouped into 26 categories according to Supplementary Table 3.

### Univariate analyses

All variables were used as predictors in three “one-vs.-the-rest” univariate logistic regression models, one for each subpopulation outcome at 24 h of post-enrollment. Continuous variables (such as height or PaO2) were standardized by dividing by the difference of the 80th and 20th quantiles. Coefficients and confidence intervals were calculated on the logit scale and exponentiated to give estimates and 95% confidence intervals for odds ratios.

### Machine learning models for outcome prediction

Twelve combinations of model architectures and frameworks were tested using R version 3.2.3. The three architectures were: gradient boosting (xgboost package, version 0.90.0.2^28^), random forest (randomForest package, version 4.6.14^29^), and logistic regression (glmnet package, version 2.0.18^30^). Each model architecture was used in four model setups: two-way (single classifier), three-way (three one-vs.-the-rest classifiers), and nested three-way (two nested classifiers), and multiclass.

The two-way setup directly modeled long-stay vs. rapid death/spontaneous recovery. Samples were classified as long-stay or short-stay (rapid death or spontaneous recovery) based on the probability output by the model. The cutoff was 0.5 for logistic regression and chosen to maximize the *F*-0.5 score for gradient boosting and random forest.

The three-way set up required fitting three one-vs.-rest classifiers (rapid death vs. long-stay/spontaneous recovery, spontaneous recovery vs. rapid death/long-stay, and long-stay vs. rapid death/spontaneous recovery). The predicted category from this setup was the category with the highest probability of the three.

The three-way nested setup fit a model to predict rapid death vs. long-stay/spontaneous recovery, and then among long-stay and spontaneous recovery patients a second model was fit to predict long-stay vs. spontaneous recovery. Rapid death was predicted if the probability for the first model was sufficiently high; if not, the predicted category corresponded to the most probable from the second model. Cutoff values for the two models were 0.5 for logistic regression, and chosen to maximize *F*-0.5 for gradient boosting and random forest.

The three-way multiclass setup modeled all three classes jointly. The model then outputs a probability for each class, with the probabilities summing to one. The predicted category was the one with highest probability. For the logistic regression architecture, a multinomial regression with a LASSO penalty was used.

For both random forest and logistic regression, mean imputation was used for missing values. Gradient boosting did not require imputation.

Hyperparameter tuning was done using five-fold cross-validation on the training dataset to choose the set of parameters that resulted in the model with the highest average accuracy across all folds. These chosen parameters were then used to train a model on the full training dataset. Separate hyperparameters were chosen for each model architecture and model setup.

The final hyperparameters for the final model (multiclass gradient boosting) are shown in Supplementary Table 4.

All available variables were used as predictors (see Supplementary Table 1). One single ward, the second largest, was completely held out for an external validation set. The remaining 47,892 ARDS ICU stays were split 70/30% into training and internal validation sets, stratified by outcome.

True positive rate (TPR), positive predictive value (PPV), and the *F*-0.5 score (a composite of TPR and PPV that weights PPV more heavily) were evaluated on the training and internal validation datasets to identify the model setup and architecture with the best performance. The *F*-0.5 score was used because it is a composite of power and positive predictive value (PPV). However, it weights PPV more heavily. This is desirable in our model building because the ability of the model to reduce the sample size needed for a trial is directly related to the PPV.

These metrics, along with AUC and calibration, were used to evaluate final model performance on the training, internal validation, and external validation samples. To calculate the AUC for the long-stay outcome from the multiclass models, a receiver operating characteristic curve was constructed using the predicted probability of a long-stay outcome output by the model for each patient. The true outcomes were dichotomized: long-stay outcome vs. rapid-death and spontaneous-recovery outcomes. Model calibration was calculated for each outcome category separately. For each outcome category (rapid death, spontaneous recovery, or long-stay), we divided the distribution of predicted probabilities into deciles. For all samples falling in each decile, the observed probability of the outcome was calculated. To summarize the predicted probability deciles, the median and range were also calculated.

Selecting a feature subset was done in one of two ways: re-training the model using only the 15 most important features (as measured by the “gain” metric), or using the Boruta feature selection method^31^.

Feature importance was evaluated using the “gain” metric implemented in xgboost.

### Sample size reduction

To calculate the expected reduction in sample size, we assumed that there is a target number (*T*) of long stay patients necessary to achieve sufficient statistical power. The total number of patients, therefore, is the target divided by the prevalence of long stay patients in the cohort: *T*/*p*_longstay_. Without a model, *p*_longstay_ is simply the prevalence of long stay patients among the ARDS population; with a model, *p*_longstay_ becomes the percentage of patients selected by the model who are long stay (the PPV). The ratio of the original sample size to the sample size needed when employing the model is therefore PPV/(long stay prevalence).

### Evaluation of prediction at identification time

We re-trained a multiclass gradient boosting model using the same input feature set and hyperparameter tuning procedure, but inputting data from the 24 h prior to identification rather than the 24 h afterwards. For this re-training, we only included patients whose ARDS began at least 6 h after ICU admission (25,087 patients). We also constructed versions of this identification-time model using Boruta-selected features, the top 15 features, and only the *P*/*F* ratio.

### Reporting summary

Further information on research design is available in the Nature Research Reporting Summary linked to this article.

## Supplementary information

Supplementary Information

Reporting Summary

## Data Availability

The data that support the findings of this study are available from the corresponding author upon reasonable request.

## Code availability

The source code for the analyses is available from the corresponding author upon reasonable request.
