# Supplementary material for: Utilizing machine learning to improve clinical trial design for acute respiratory distress syndrome
Source: NPJ Digit Med. 2021 Sep 9;4:133. doi: 10.1038/s41746-021-00505-5 (PMC8429640; doi:10.1038/s41746-021-00505-5)
Supplement: Supplementary file 1 — Supplementary Information [file 41746_2021_505_MOESM1_ESM.pdf]

Supplement

Supplementary Tables

Name	Type	Name	Type	Name	Type
Admission diagnosis	Static	FiO2	Dynamic	Indicator of norepinephrine use	
Admission source	Static	GCS (eyes)	Dynamic	PaCO2	Dynamic
Age	Static	GCS (intub)	Dynamic	PaO2	Dynamic
Albumin	Dynamic	GCS (total)	Dynamic	PAP (diastolic)	Dynamic
ALP	Dynamic	GCS (motor)	Dynamic	PAP (mean)	Dynamic
ALT	Dynamic	GCS (unable)	Dynamic	PAP (systolic)	Dynamic
Indicator of any vasopressor use		GCS (verbal)	Dynamic	P/F ratio	Dynamic
APACHE IV Score at admission	Static	Gender	Static	PIP	Dynamic
AST	Dynamic	Glucose	Dynamic	PEEP	Dynamic
Bands	Dynamic	Heart rate	Dynamic	Indicator of phenylephrine use	
Base excess	Dynamic	Hematocrit	Dynamic	pH	Dynamic
Basos	Dynamic	Hemoglobin	Dynamic	Platelets	Dynamic
Bicarbonate	Dynamic	Indicator of heparin use		Plateau pressure	Dynamic
Bilirubin	Dynamic	INR	Dynamic	Potassium	Dynamic
BMI	Static	Invasive BP (diastolic)	Dynamic	PTT	Dynamic
BUN	Dynamic	Invasive BP (mean)	Dynamic	SaO2	Dynamic
Calcium	Dynamic	Invasive BP (systolic)	Dynamic	Sodium	Dynamic
CO2	Dynamic	Ionized calcium	Dynamic	SpO2	Dynamic
Creatinine	Dynamic	Lactate	Dynamic	Respiration rate	Dynamic
CVP	Dynamic	Magnesium	Dynamic	Temperature	Dynamic
Indicator of dobutamine use		Mean airway pressure	Dynamic	Tidal volume/IBW	Dynamic
Indicator of dopamine use		Indicator of milrinone use		Indicator of vasopressin use	
Indicator of epinephrine use		NIBP (diastolic)	Dynamic	Indicator of warfarin use	
Eos	Dynamic	NIBP (mean)	Dynamic	WBC	Dynamic
etCO2	Dynamic	NIBP (systolic)	Dynamic		

Table 1: *Indicators are 1 if the drug was administered in the 24h after the identification time and 0 otherwise. Admission diagnosis and admission source have 26 and 5 unique values, respectively.*

Name	Limit
Albumin	[0, 7]
ALP	[0,550]
ALT	[7,1000]
Bicarbonate	[5,40]
CVP	[0, 100]
FiO2	[20, 100]
heartrate	[30,175]
INR	[0,15]
Lab_AST	[10,1000]
Lab_Bands	[0,70]
Lab_Blood_Urea_Nitrogen	[0,115]
Lab_Calcium	[0,50]
Lab_CO2	[10,45]
Lab_Creatinine	[0,8]
Lab_Glucose	[0,500]
Lab_Hematocrit	[14,55]
Lab_Hemoglobin	[0,16]
Lab_Ionized_Calcium	[0, 10]
Lab_Lactate	[0,15]
Lab_Magnesium	[0, 10]
Lab_PaCO2	[10,100]
Lab_Potassium	[0,12]
Lab_PTT	[20,160]
Lab_SaO2	[80,100]

Name	Limit
Lab_Sodium	[95,215]
Lab_Total_Bilirubin	[0,20]
Lab_WBC	[0,60]
Mean_Airway_Pressure	[5,30]
nonInvasiveDiastolic	[30,150]
nonInvasiveMean	[30,150]
nonInvasiveSystolic	[30,250]
PaO2	(0,650]
PAPDia	(0,40]
PAPMean	(0,60]
PAPSys	(0,80]
PEEP	[0,25]
Peak_Insp_Pressure	[-Inf, 1000]
P/F ratio	(0, 650]
pH	[7,7.7]
Platelets	[0,1000]
PPlat	[4,50]
respiration	[1,40]
SpO2	[80,100]
systemicDiastolic	[30,150]
systemicMean	[30,150]
systemicSystolic	[30,250]
Temperature	[30,45]
TVInsp	[-Inf, 1000]

Supplementary Table 2: The ranges used to remove outliers for continuous features.

Group	Diagnoses
Acute Coronary Syndrome	“Angina, unstable (angina interferes w/quality of life or meds are tolerated poorly)”
Acute Myocardial Infarction	“Infarction, acute myocardial (MI)”
	“MI admitted > 24 hrs after onset of ischemia”
Acute Renal Failure	“Renal failure, acute”
	“Renal obstruction”
Arrhythmia	“Ablation or mapping of cardiac conduction pathway”
	“Defibrillator, automatic implantable cardiac, insertion of”
	“Rhythm disturbance (atrial, supraventricular)”
	“Rhythm disturbance (conduction defect)”
	“Rhythm disturbance (ventricular)”
Asthma or Emphysema	“Asthma”
	“Emphysema/bronchitis”
Cancer	“Cancer, colon/rectal”
	“Cancer, esophageal”
	“Cancer, laryngeal”
	“Cancer, lung”
	“Cancer, oral”
	“Cancer, oral/sinus, surgery for”
	“Cancer, other GI”
	“Cancer, pancreatic”
	“Cancer, stomach”
	“Cancer, tracheal”
	“Cancer-colon/rectal, surgery for (including abdominoperineal resections)”
	“Cancer-esophageal, surgery for (abdominal approach)”
	“Cancer-laryngeal/tracheal, surgery for”
	“Cancer-other GI tract, surgery for (ie., hepatoma, gallbladder etc.)”
	“Cancer-small intestinal, surgery for”
	“Cancer-stomach, surgery for”
	“Leukemia, acute lymphocytic”
	“Leukemia, acute myelocytic”
	“Leukemia, chronic lymphocytic”
	“Leukemia, chronic myelocytic”
	“Leukemia, other”
Cardiac Arrest	“Cardiac arrest (with or without respiratory arrest, for respiratory arrest see Respiratory System)”

Group	Diagnoses
Cardiogenic Shock	“Shock, cardiogenic”
Cardiovascular (Medical)	“Cardiovascular medical, other”
Cardiovascular (Other)	“Angina, stable (asymp or stable pattern of symptoms w/meds)”
	“Anomaly, cardiac congenital", "Arteriovenous malformation, surgery for”
	“Atrial Septal Defect (ASD) Repair”
	“Cardiovascular surgery, other”
	“Congenital Defect Repair (Other)”
	“CVOther =\"Contusion, myocardial (include r/o)”
	“Efffusion, pericardial”
	“Endocarditis”
	“Hypertension-pulmonary, primary/idiopathic”
	“Monitoring, hemodynamic (pre-operative evaluation)”
	“Pericardial effusion/tamponade”
	“Pericardiectomy (total/subtotal)”
	“Pericarditis”
	“Tamponade, pericardial”
	“Thrombus, arterial”
	“Vascular medical, other”
	“Vascular surgery, other”
Cerebrovascular Accident/Stroke	“CVA, cerebrovascular accident/stroke”
	“Hemorrhage/hematoma, intracranial”
	“Hemorrhage/hematoma-intracranial, surgery for”
	“Hypertension, uncontrolled (for cerebrovascular accident-see Neurological System)”
	“Subarachnoid hemorrhage/arteriovenous malformation”
	“Subarachnoid hemorrhage/intracranial aneurysm”
	“Subarachnoid hemorrhage/intracranial aneurysm, surgery for”
Chest Pain Unknown Origin	“Chest pain, atypical (noncardiac chest pain)”
	“Chest pain, epigastric”
	“Chest pain, musculoskeletal”
	“Chest pain, respiratory”
	“Chest pain, unknown origin”
Coma	“Coma/change in level of consciousness (for hepatic see GI, for diabetic see Endocrine, if related to cardiac arrest, see CV)”
	“Nontraumatic coma due to anoxia/ischemia”

Supplementary Table 3A: Diagnosis groupings.

Group	Diagnoses
Coronary Artery Bypass Graft	“CABG alone, coronary artery bypass grafting”
	“CABG alone, redo”
	“CABG redo with other operation”
	“CABG redo with valve repair/replacement”
	“CABG with aortic valve replacement”
	“CABG with double valve repair/replacement”
	“CABG with mitral valve repair”
	“CABG with mitral valve replacement”
	“CABG with other operation”
	“CABG with pulmonic or tricuspid valve repair or replacement ONLY.”
	“CABG with single valve repair/replacement”
	“CABG, minimally invasive, mid-CABG”
Diabetic Ketoacidosis	“Diabetic hyperglycemic hyperosmolar nonketotic coma (HHNC)”
	“Diabetic ketoacidosis”
Gastrointestinal Bleed	“Bleeding, GI from esophageal varices/portal hypertension”
	“Bleeding, GI-location unknown”
	“Bleeding, lower GI”
	“Bleeding, upper GI”
	“Bleeding-lower GI, surgery for”
	“Bleeding-other GI, surgery for”
	“Bleeding-upper GI, surgery for”
	“Bleeding-variceal, surgery for (excluding vascular shunting-see surgery for portosystemic shunt)”
	“GI perforation/rupture”
	“GI perforation/rupture, surgery for”
	“Hemorrhage, intra/retroperitoneal”
	“Ulcer disease, peptic”
Gastrointestinal Obstruction	“GI obstruction”
	“GI obstruction, surgery for (including lysis of adhesions)”
Neurologic	“Abscess, neurologic”
	“Biopsy, brain”
	“Hydrocephalus, obstructive”
	“Neoplasm, neurologic”
	“Neoplasm-cranial, surgery for (excluding transphenoidal)”
	“Neoplasm-spinal cord, surgery or other related procedures”
	“Neurologic medical, other”
	“Neuromuscular medical, other”
	“Palsy, cranial nerve”
	“Seizures (primary-no structural brain disease)”
	“Seizures-intractable, surgery for”
Overdose	“Overdose, alcohols (bethanol, methanol, ethylene glycol)”
	“Overdose, analgesic (aspirin, acetaminophen)”

Group	Diagnoses
Overdose	“Overdose, antidepressants (cyclic, lithium)”
	“Overdose, other toxin, poison or drug”
	“Overdose, sedatives, hypnotics, antipsychotics, benzodiazepines”
	“Overdose, self-inflicted”
	“Overdose, street drugs (opiates, cocaine, amphetamine)”
	“Toxicity, drug (i.e., beta blockers, calcium channel blockers, etc.)”
Pneumonia	“Pneumonia, aspiration”
	“Pneumonia, bacterial”
	“Pneumonia, fungal”
	“Pneumonia, other”
	“Pneumonia, parasitic (i.e., Pneumocystic pneumonia)”
	“Pneumonia, viral”
Respiratory (Medical/Other)	“Apnea, sleep”
	“Apnea-sleep, surgery for (i.e., UPPP - uvulopalatopharyngoplasty)”
	“ARDS-adult respiratory distress syndrome, non-cardiogenic pulmonary edema”
	“Arrest, respiratory (without cardiac arrest)”
	“Atelectasis”
	“Biopsy, open lung”
	“Effusions, pleural”
	“Embolus, pulmonary”
	“Guillain-Barre syndrome”
	“Hemorrhage/hemoptysis, pulmonary”
	“Hemothorax”
	“Obstruction-airway (i.e., acute epiglottitis, post-extubation edema, foreign body, etc)”
	“Pneumothorax”
	“Respiratory - medical, other”
	“Restrictive lung disease (i.e., Sarcoidosis, pulmonary fibrosis)”
	“Tracheostomy”
	“Weaning from mechanical ventilation (transfer from other unit or hospital only)”
Sepsis	“Sepsis, cutaneous/soft tissue”
	“Sepsis, GI”
	“Sepsis, gynecologic”
	“Sepsis, other”
	“Sepsis, pulmonary”
	“Sepsis, renal/UTI (including bladder)”
	“Sepsis, unknown”
Thoracotomy	“Thoracotomy for benign tumor (ie. mediastinal chest wall mass, thymectomy)”
	“Thoracotomy for bronchopleural fistula”
	“Thoracotomy for esophageal cancer”
	“Thoracotomy for lung cancer”
	“Thoracotomy for lung reduction”

Supplementary Table 3B: Diagnosis groupings.

Group	Diagnoses
Thoracotomy	"Thoracotomy for other malignancy in chest"
	"Thoracotomy for other reasons"
	"Thoracotomy for pleural disease"
	"Thoracotomy for thoracic/respiratory infection"
Trauma	"Abdomen only trauma"
	"Abdomen/extremity trauma"
	"Abdomen/face trauma"
	"Abdomen/multiple trauma"
	"Abdomen/pelvis trauma"
	"Abdomen/spinal trauma"
	"Chest thorax only trauma"
	"Chest/abdomen trauma"
	"Chest/extremity trauma"
	"Chest/face trauma"
	"Chest/multiple trauma"
	"Chest/pelvis trauma"
	"Chest/spinal trauma"
	"Chest/thorax only trauma"
	"Extermity only trauma"
	"Extremity only trauma, surgery for"
	"Extremity/face trauma"
	"Extremity/face trauma, surgery for"
	"Extremity/multiple trauma"
	"Extremity/multiple trauma, surgery for"
	"Face only trauma"
	"Face only trauma, surgery for"
	"Face/multiple trauma"
	"Face/multiple trauma, surgery for"
	"Facial surgery (if related to trauma, see Trauma)"
	"Head only trauma"

Group	Diagnoses
Trauma	"Head/abdomen trauma"
	"Head/chest trauma"
	"Head/extremity trauma"
	"Head/face trauma"
	"Head/multiple trauma"
	"Head/pelvis trauma"
	"Head/spinal trauma"
	"Pelvis/extremity trauma"
	"Pelvis/face trauma"
	"Pelvis/hip trauma"
	"Pelvis/multiple trauma"
	"Pelvis/spinal trauma"
	"Spinal cord only trauma"
	"Spinal/extremity trauma"
	"Spinal/face trauma"
	"Spinal/multiple trauma"
	"Trauma medical, other"
	"Trauma surgery, other"
Valve Disease	"Aortic and Mitral valve replacement"
	"Aortic valve replacement (isolated)"
	"Mitral valve repair"
	"Mitral valve replacement"
	"Papillary muscle rupture"
	"Pulmonary valve surgery"
	"Tricuspid valve surgery"
	"Valve, double, repair/replacement"
	"Valve, redo, single"
	"Valve, single, repair/replacement"
	"Valve, triple, repair/replacement"

Supplementary Table 3C: Diagnosis groupings.

Hyperparameter	Value
Eta	0.3
Gamma	10
Maximum depth	6
Minimum child weight	1
Number of iterations	25

Supplementary Table 4: *The hyperparameter values selected by 5-fold cross-validation for the final multiclass gradient boosting model*

Cohort	ECI Grouped Score	Brier Score	Maximum Brier Score
Train	0.229	0.126	0.513
Internal Validation	0.060	0.135	0.513
External Validation	0.897	0.125	0.435

Supplementary Table 5: Two calibration metrics for each cohort. *An adjusted ECI score using ten bins (1) and the Brier score compared with the maximum possible based on the outcome prevalences in each cohort (2).*

**Cohort**		**H-L p-value**
Train	Long stay vs. others	< 0.001
	Rapid death vs. others	< 0.001
	Spontaneous recovery vs. others	< 0.001
Internal Validation	Long stay vs. others	< 0.001
	Rapid death vs. others	< 0.001
	Spontaneous recovery vs. others	< 0.001
External Validation	Long stay vs. others	< 0.001
	Rapid death vs. others	0.442
	Spontaneous recovery vs. others	< 0.001

Supplementary Table 6: The p-value for the Hosmer-Lemeshow test for each calibration curve. *Ten groups were used for each test.*

Supplementary Figures

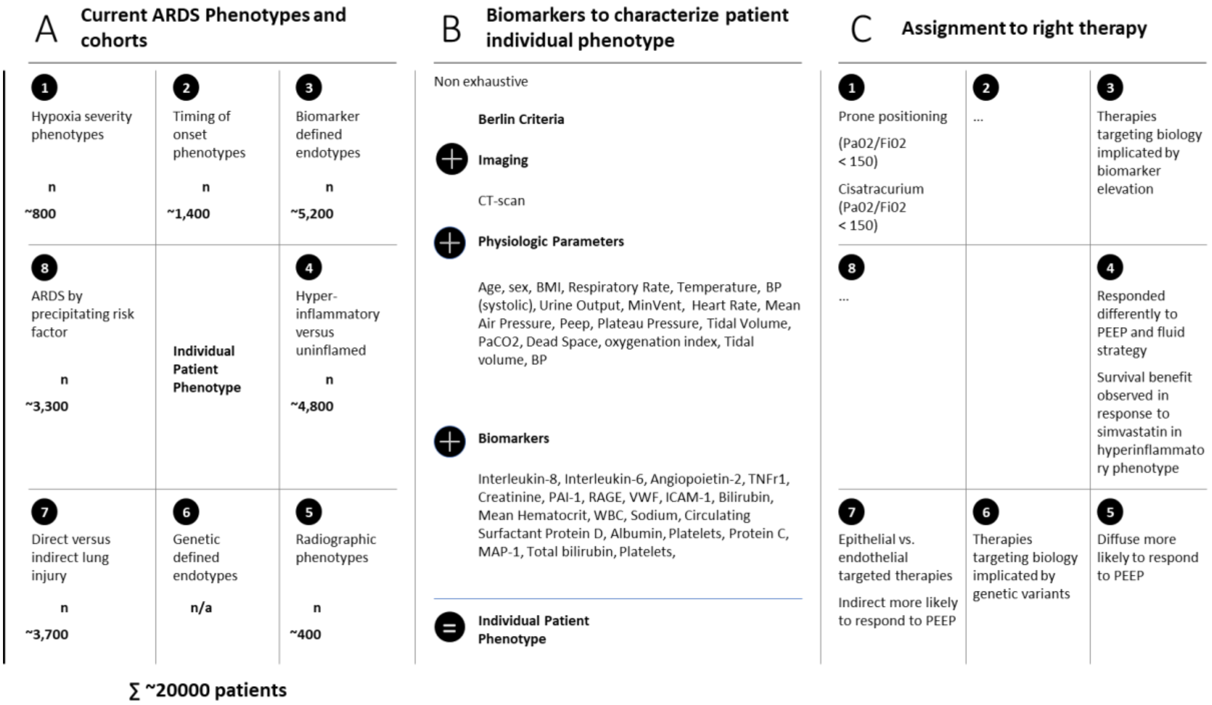

Supplementary Figure 1: Current phenotypes of ARDS patients according to Reilley et al. (hier nochmal die Nummer aus dem Paper) and respective interventions. *(**A**) Currently, eight different ARDS phenotypes exist based on around 20.000 cases that have been investigated to create predictive phenotypes. (**B**) However, clinically characterizing patients to fit one of these phenotypes is challenging, takes time, and involves different examinations. (**C**) Despite it has been shown that characterizing patients provides a benefit with a view on distinctive treatments (Data derived from Rilley et al., Semin Respir Crit Care Med. 2019;40(1):19-30)*

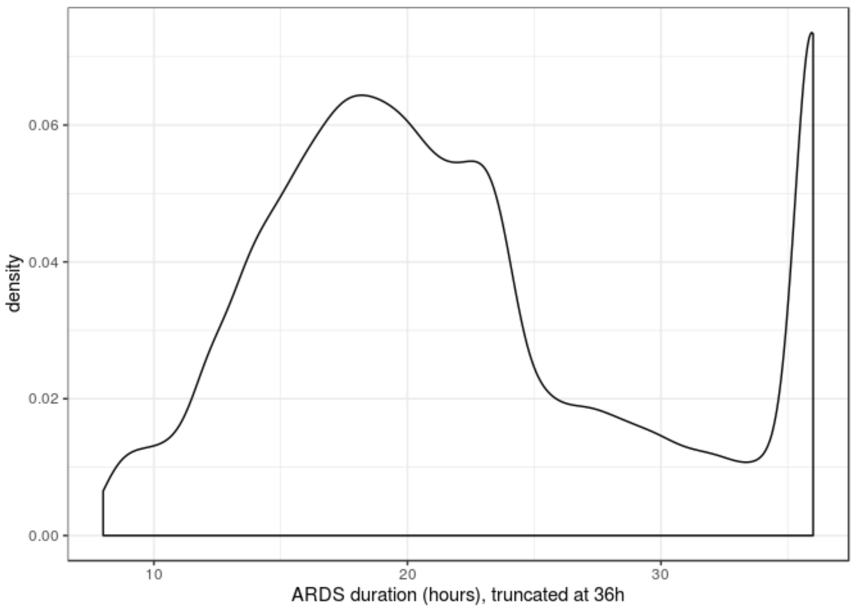

Supplementary Figure 2: Duration of ARDS episodes across the cohort of ARDS patients. *The duration of all detected ARDS episodes is calculated, and durations greater than 36 hours are replaced with 36 hours.*

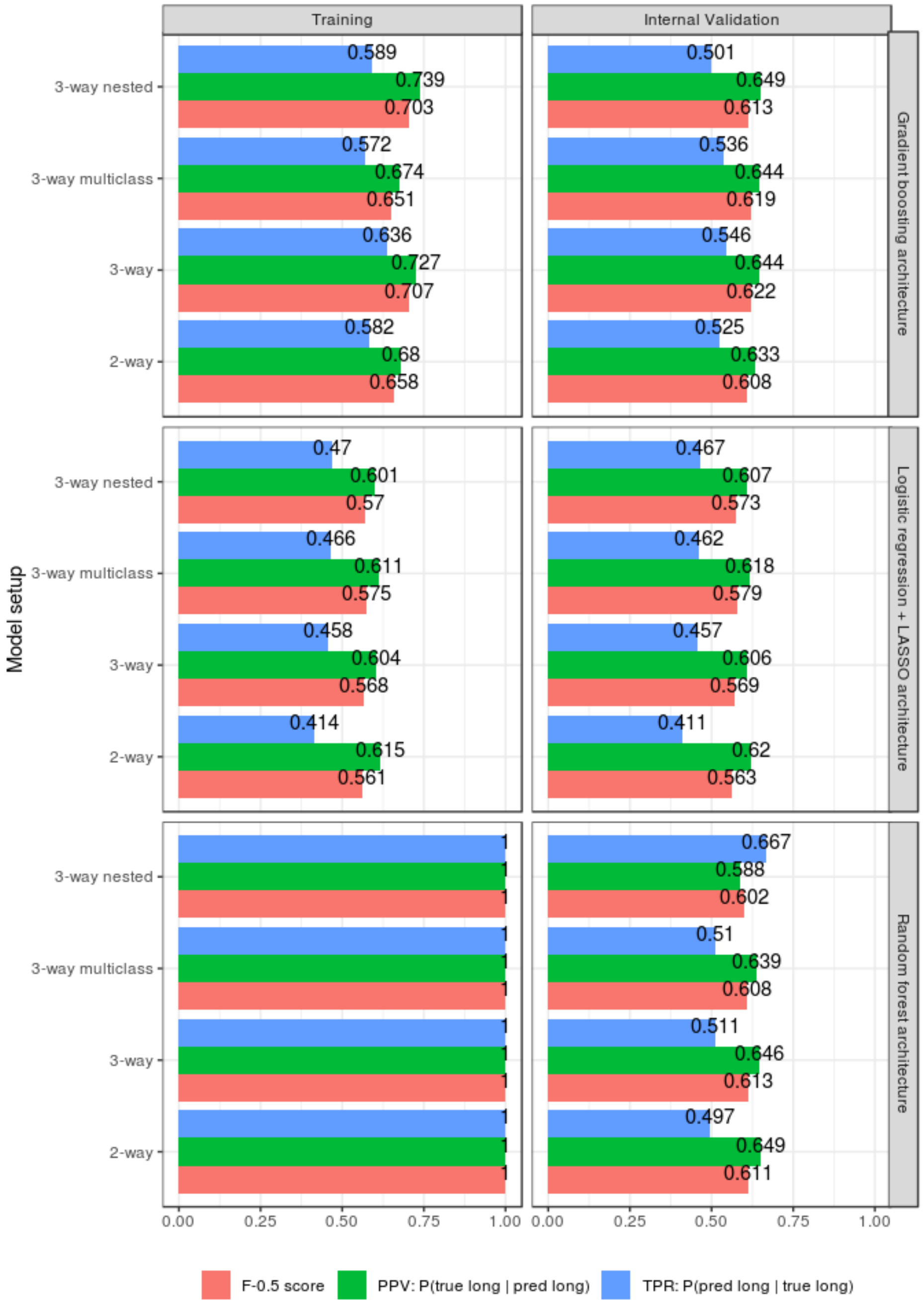

Supplementary Figure 3: Performance of all models evaluated. *Performance of all trained models on both the training and internal validation sets. Performance is evaluated using the PPV (positive predictive value), TPR (true positive rate), and F-0.5 score, a composite score of the PPV and TPR.*

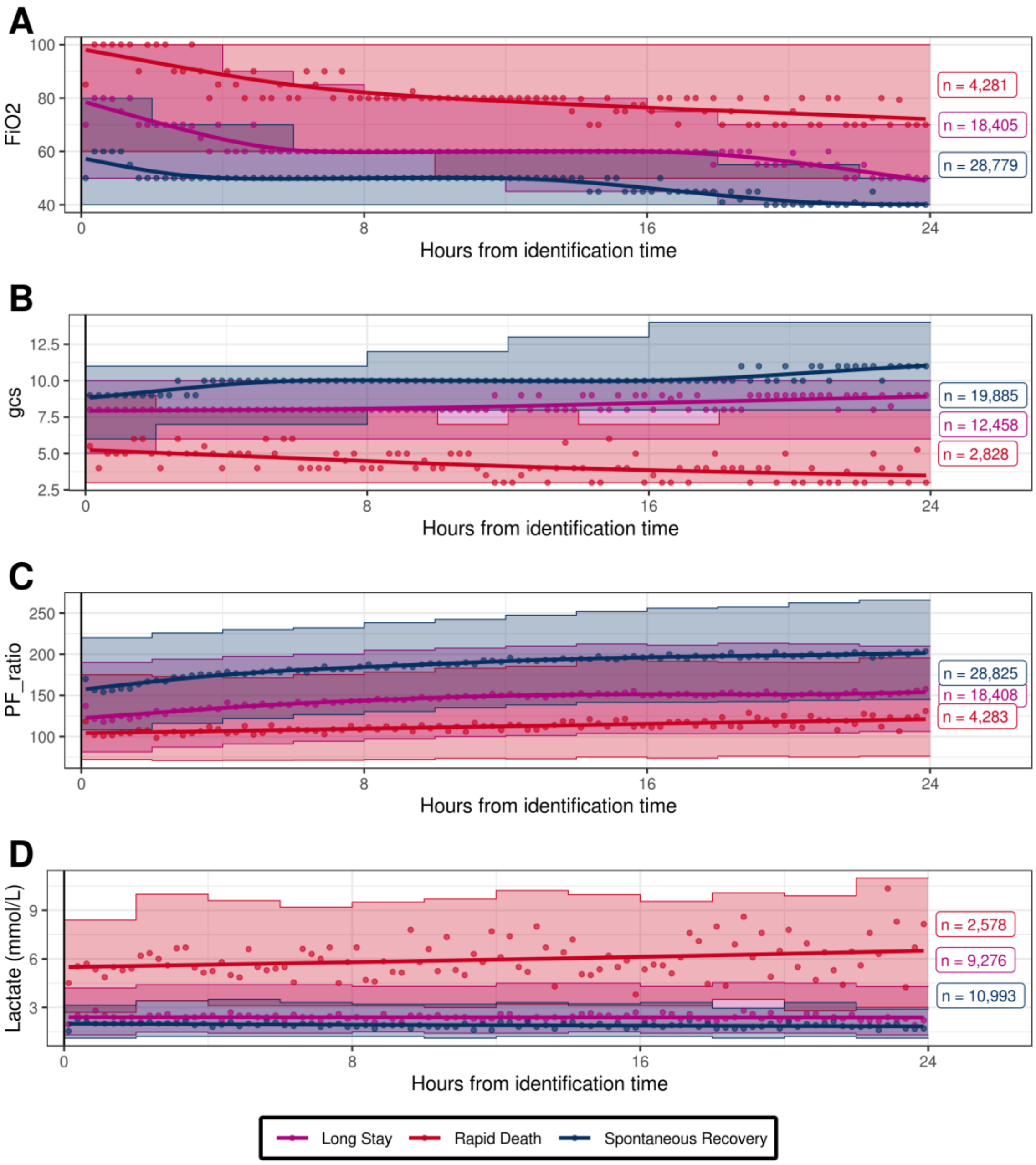

Supplementary Figure 4: Time course plots of the top four continuous features from the multiclass gradient boosting model. *Smoothed trends between the identification time and 24 hours afterwards by patient subtype for FiO2 (**A**), GCS (**B**), P/F ratio (**C**), and lactate (**D**). Points represent the median parameter values in a quarter-hour interval relative to identification time. Intervals represent the 25th and 75th percentiles of parameter values in a two-hour window relative to identification time. GAM smoothing splines highlight trends. Number of subjects with plausible values in each category is shown on the right of each plot.*

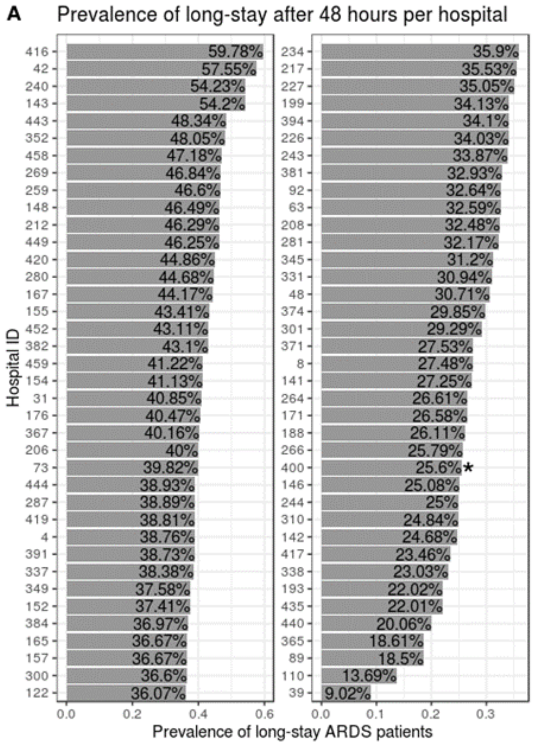

Supplementary Figure 5: Prevalence of long-stay patients among those alive at enrolment by hospital. *Percentage of ARDS patients alive 24 hours after identification (at enrolment) who experience continue to have ARDS events 48 hours after identification. Hospitals with fewer than 100 ARDS patients alive at enrolment are not shown. The asterisk (*) indicates the hospital containing the ward used for the external validation set.*

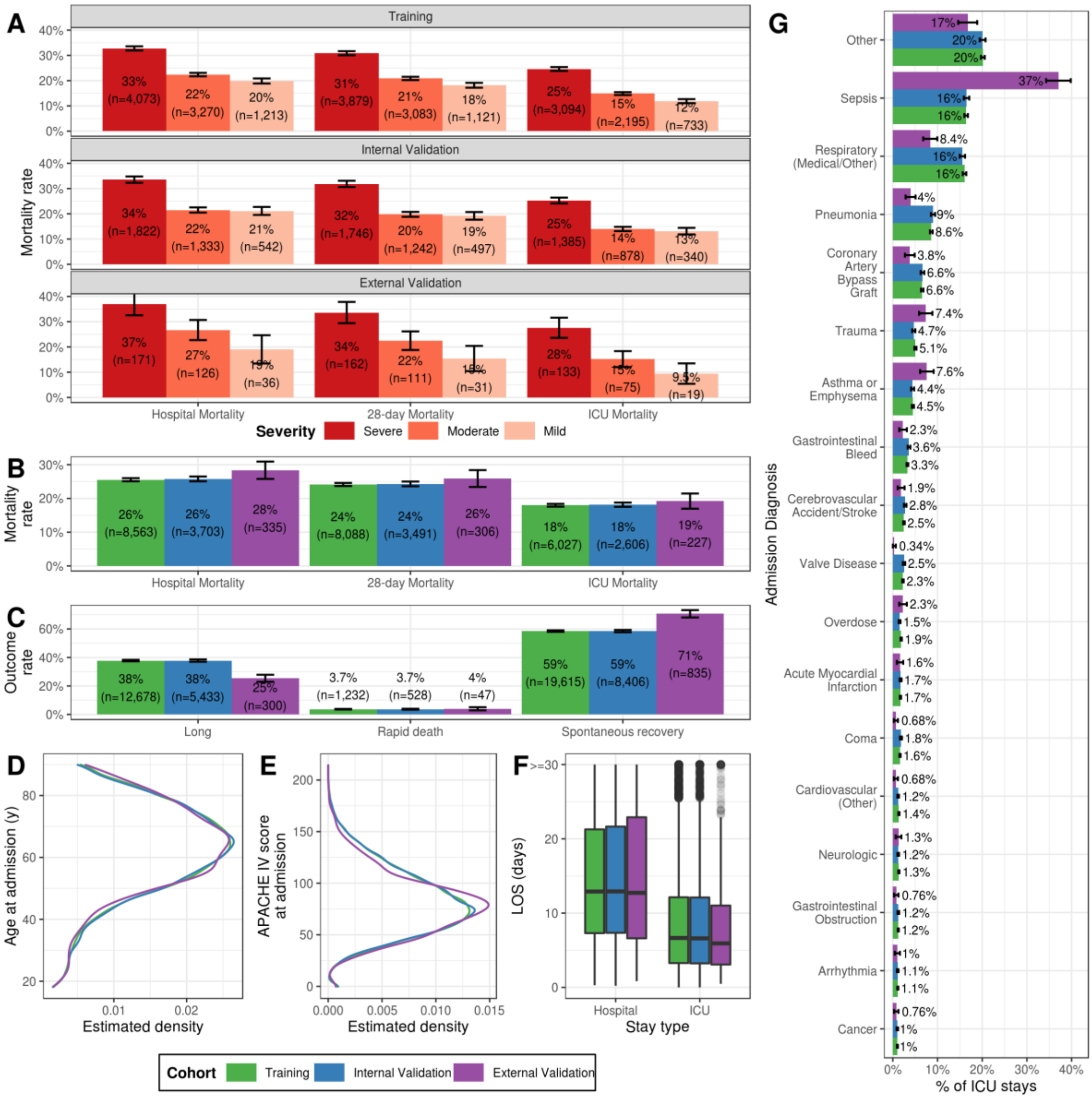

Supplementary Figure 6: Demographics and outcomes among training, internal validation, and external validation sets. *The mortality rate by severity at onset (**A**), overall mortality rates (**B**), outcome rates (**C**) age (**D**), APACHE IV scores (**E**), lengths of stay (LOS) both in-hospital and in-ICU (**F**), and admission diagnoses (**G**). Severity was determined by the minimum P/F ratio in the first 8 hours after ARDS onset. Error bars represent 95% confidence intervals. Panels (**D**) and (**E**) represent estimated densities. In panel (**F**), the boxplot elements are: center line, median; box limits, upper and lower quartiles; whiskers, 1.5x interquartile range; points, outliers.*

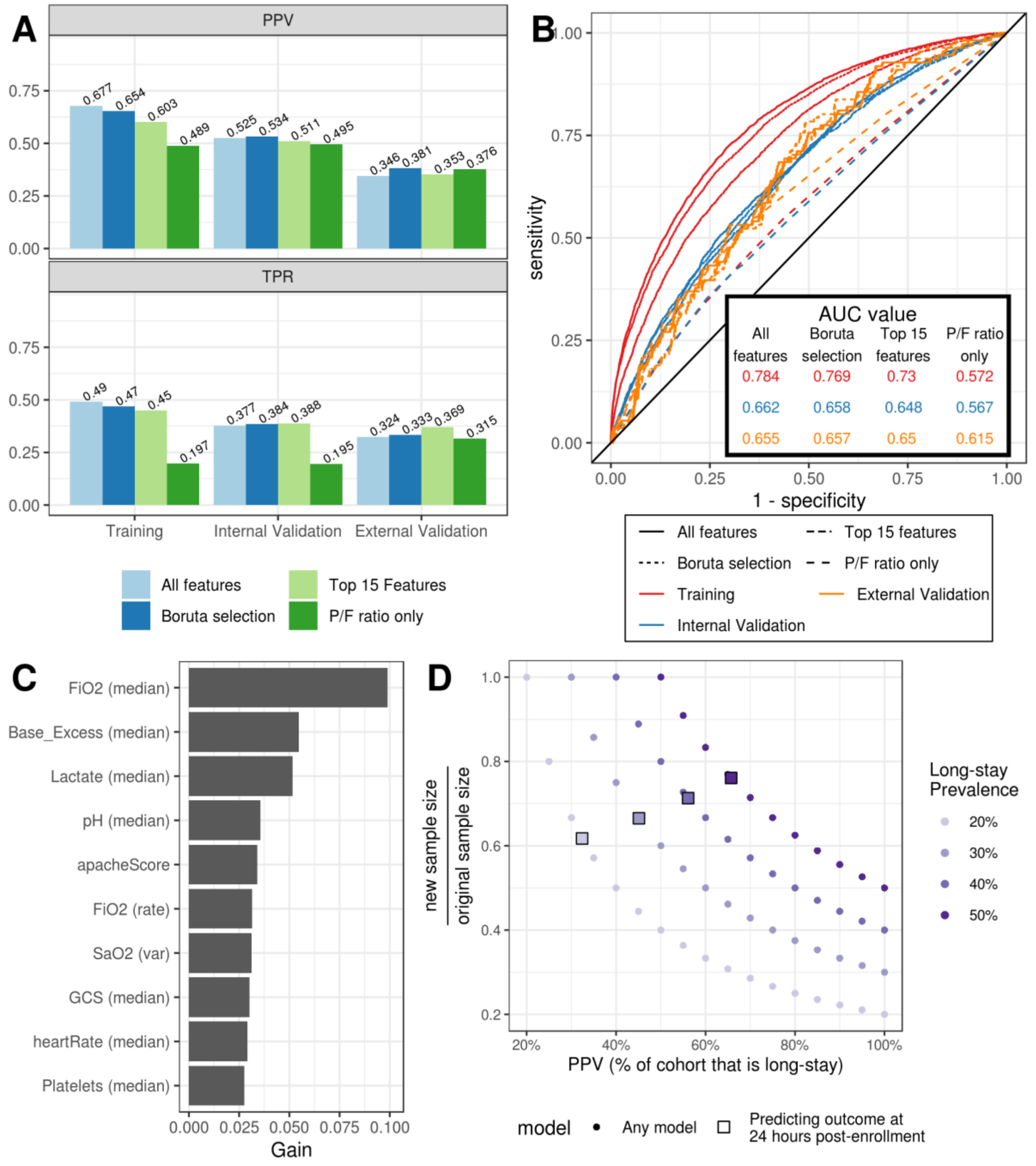

Supplementary Figure 7: Predicting patients with a long ARDS course at identification time. *(**A**) The performance of the identification-time model on the internal and external validation datasets as measured by the positive predictive value (PPV) and true positive rate (TPR). (**B**) The receiver operating characteristic curves for identifying long stay vs. other patients; see **Methods**. (**C**) The top 10 features by gain are shown (GCS=Glasgow coma scale). (**D**) The effect of the model on total sample size. Panels (**C**)-(**D**) display results from the Boruta-selected identification-time model.*

References

1. Van Hoorde K, Van Huffel S, Timmerman D, Bourne T, Van Calster B. A spline-based tool to assess and visualize the calibration of multiclass risk predictions. J Biomed Inform. 2015;54:283-93.

2. Steyerberg EW, Vickers AJ, Cook NR, Gerds T, Gonen M, Obuchowski N, et al. Assessing the performance of prediction models: a framework for traditional and novel measures. Epidemiology. 2010;21(1):128-38.
